# Methods Development for the Constrained Elastic Modulus Investigation of Organic Material in Natural Soil Conditions

**DOI:** 10.3390/ma14226842

**Published:** 2021-11-12

**Authors:** Zygmunt Meyer, Magdalena Olszewska

**Affiliations:** Faculty of Civil and Environmental Engineering, West Pomeranian University of Technology Szczecin, Al. Piastów 17, 70-310 Szczecin, Poland; meyer@zut.edu.pl

**Keywords:** constrained modulus, modulus of elasticity, peat, compressibility of organic soil

## Abstract

Compressibility is one of the most important mechanical properties of soil. The parameter that characterizes compressibility is the constrained modulus of elasticity. Knowledge of this is important to calculate the settlement of a structure foundation on peat material. According to soil classification by EN ISO 14688-2, peat is an organic soil that contains min. 20% organic matter. It is a highly organic type of soil. Peat material has large compressibility. The value of the constrained elasticity modulus for peat is ca. 400 kPa, while it may be ca 1.0–1.6 MPa for consolidated peat. Due to the extensive range of the modulus, experimental research in this field is proposed. It is suggested to load the peat material layer with an embankment and to determine its total settlement. Based on this, a program was developed to determine the settlement–strain relationship. The authors propose an approach according to two models: the first is based on constant stress distribution in the soil with an oedometer test. The second considers the variability of stresses in the soil and the influence of the loaded area. Both methods were tested based on numerical simulations, and then an experimental field in Szczecin was used. The formulae for the constrained modulus of elasticity measurement were derived; in practical conditions, a uniaxial deformation state can be used with the combination of the total settlement.

## 1. Introduction

Peat material is a highly organic soil. It comprises decomposed remains of plants in a wet environment [1,2,3]. Peat has a dark brown to black colour, spongy consistency, and characteristic organic odour [2]. Many behavioural parameters and properties are similar to clay soils, but they are radically different from strength characteristics. Therefore, it is taken for an immensely soft soil [3,4]. Peat has a high water content. In this soil, water can comprise 90% of the mass; thus, it is a poor substrate for buildings [1,4]. The natural moisture content can reach up to 1500%. This type of soil has low density and organic matter contents. Unit weight for this type of soil is 8 to 14.3 kN m^−3^ [2,5]. Huge water content (>200%) and porosity are also reasons for huge compressibility of peat (including significant secondary and tertiary compression), low stiffness and low shear strength (ca. 5–20 kPa) [2,6,7,8]. Peat also shows a tendency to creep [7]. Cohesion for this type of soil is ca. 1 to 14 kPa, and the angle of internal friction is from ca. 3 to 24 [2,9]. Other characteristic features can include high spatial variability and permeability [2]. Due to the high level of groundwater and peat’s low density, the effective tensions are relatively low [7], which shows peat has weak geotechnical properties [8].

It is crucial to identify an organic soil parameter. In comparison with mineral soil, these parameters vary. Therefore, traditional methods of soil mechanical parameters testing may not be suitable for organic material such as peat.

It is possible to choose the parameters of the compressibility of peat such that they relate to the technical parameters. However, there is an example of engineering projects to accept peat as a soil in roads, bridges and harbours. One such example, as described by Tan [10], is part of the US Route 44 relocation project. This route covers areas of cranberry bogs with deep layers of peat. Peat in the planned roadside was dug entirely and was covered with granular soils, using sheet piling as retaining structures. The leftover peat was the area of cooperation between the organic substrate and the structure. In addition, peat can be used as steel slag-dredged soil mixtures for construction materials at harbours [11]. For this reason, it is important to know the mechanical parameter of organic soil, such as the constrained modulus of elasticity.

Problems of soil materials are statically indeterminate due to stress–strain features, which are not linear. The behaviour of soil depends on the environment, time, pressure and temperature. Soil is different in every location. Physical and mechanical properties are evaluated based on small samples received from subsoil in nearly all cases. The structure of the soil is susceptible and can be disturbed by sampling. This may be a reason for the differences in measured laboratory tests versus in situ tests [12].

**Figure 1:**
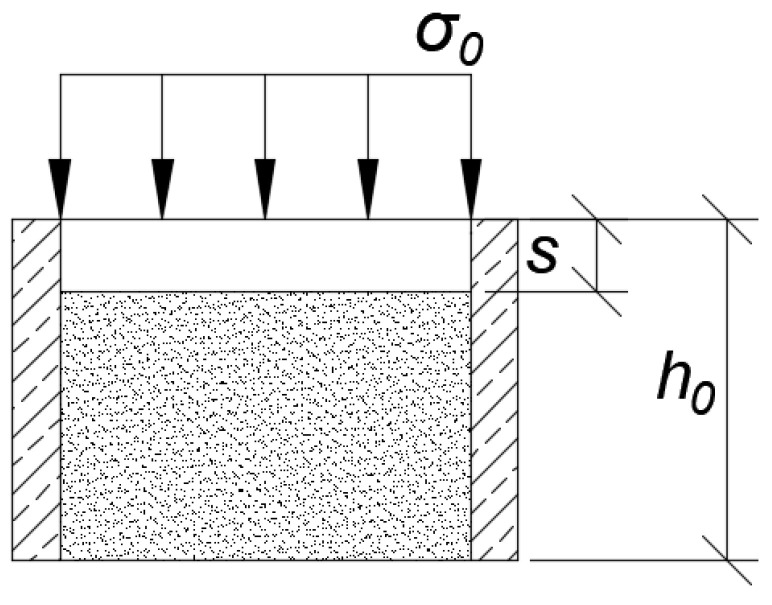
Scheme of confined compression as in oedometer test; *σ_0_*, compressive load on the soil sample (kPa); *s*, settlement of the soil sample (m); *h_0_*, primary thickness of soil sample (m).

One of the main features of the settlement calculation is the relationship between stress and strain [7]. The stress–strain behaviour is complex, and depends on the composition, void ratio, and method of stress applied. In analyses, this behaviour uses formulae and concepts from the theory of elasticity, which is simplified because the soil’s non-linear curve of stress–strain relation is replaced by a straight line [12]. Sometimes, in stress–strain relationships, it is convenient to use a soil modulus. This modulus will be different for each different type of soil. This parameter is not constant for soil but is a parameter that describes the behaviour of soil for a given set of stresses [12]. The constrained modulus of elasticity describes soil deformation under the influence of a given load in a uniaxial state without the possibility of lateral deformation. This type of modulus is shown in Figure 1.

The constrained modulus of elasticity *E* is one of the major compressibility parameters. It is used in the one-dimensional analysis of consolidation [7]. This parameter is an inverse of the coefficient of volume compressibility (*m_v_*). The modulus is the parameter that describes uniaxial stress to uniaxial strain for limited compression [12]. This parameter is highly dependent on pressure level [13].

The large compressibility of peat characterizes organic soil. Therefore, deformation characteristics are significant. Final settlement under a given load may take a long time due to the mechanical properties of organic soil. Therefore, long-term consolidation processes result in considerable deformation [2]. Compared to other soils, peat compression takes much longer: the primary consolidation is swift; however, secondary compression takes a long time. Factors influencing peat compressibility are fibre content, natural water content, initial permeability, void ratio, and nature and arrangement of soil particles [2].

These parameters are difficult to maintain during an extended peat test in the laboratory. Engineers to calculate the settlement have to know the constrained modulus of elasticity, which is generally defined from the oedometer test, named oedometer modulus *M*_0_ (kPa) [2,14]. The settlement parameters of peat can be determined from standard oedometer (one-dimensional compression) tests similar to in mineral soils [13], but there may be some differences in findings. In the oedometer test, the soil sample is placed into a metal ring. The height of the metal ring depends on the apparatus, which is generally a few centimetres. Then, the sample is placed in an oedometer, and it is loaded by force. The value of the odometer modulus depends on the type of soil. For example, mineral soil such as sand has the biggest value on the oedometer modulus, i.e., ca. from 40,000 kPa for fine sand to 20,000 kPa [15] for coarse sand. For soils such as clay and silt, the oedometer modulus can be from 10,000 to 60,000 kPa [15]. The smallest value is for organic soil. In an oedometer, compression is a significant source of strain [12]. Stress is adhibited to the soil sample along the vertical axis without the possibility of strain in the horizontal direction. In this case, the axial strain is equal to the volumetric strain, e.g., in Figure 1 [12]. It influences the value of settling the sample in the oedometer and then the value of the oedometer modulus.

Oedometric modulus *M_0_* can be calculated from the oedometer test with Equation (1):

(1) $$M_{0}=\frac{\Delta \sigma_{i}}{\varepsilon }$$

where

*M_0_*—oedometer constrained modulus of elasticity in the range of loads from *σ_i_* to *σ_i+1_* (kPa);

Δ*σ_i_*—stress increment (kPa);

*ε*—unit deformation of the sample [12].

The constrained modulus of elasticity for peat and other organic soils is low. In Mazury lake district (Poland, Central Europe), the oedometric modulus is between 498–846 kPa for a load range from 0 to 400 kPa [16]. Near Konstancin (Poland, Central Europe), it is from 1660 to 1726 kPa [16], and near Warsaw (Poland, Central Europe), it is from 100 to 770 kPa for a load range from 0 to 100 kPa [16]. In West Pomerania (Poland, Central Europe), it is from 199 to 980 kPa for a load range from 25 to 50 kPa [17,18] and from 180 to 600 kPa for a load range from 50 to 100 kPa [17,18], and from 600 to 1570 kPa for a load range from 100 to 200 kPa [17,18]. Compared with mineral soil, these values are incredibly small.

As the load increases, organic soils show a non-linear nature of the stress–strain relationship. Even for low loads, the settlements are considerable. The constrained modulus of elasticity of loaded peat tends to increase with increasing load over time. The compressibility of peat is considerable [7,19,20]. In research by the Warsaw University of Life Sciences and the Swedish Geotechnical Institute [7,19], the variability of the compressibility modulus for the stress range was given for peat as *M*_0_ *=* 30*σ’_p_*, where *M*_0_ is the oedometric modulus and *σ’_p_* is the initial pre-consolidation pressure.

Soil can also be the basis for agriculture activity, for both construction and environmental purposes [21]. In each of these cases, vertical displacement of the crop yield may be significant, followed by the formation of porosity and by settlement of the soil due to the reduction of porosity [21]. With this in mind, the authors decided that an essential element to determine the susceptibility to organic soil load is the peat-constrained modulus of elasticity.

Most of the peat in Poland (Central Europe) is near Szczecin, where the experiment was carried out. This results in the need to strengthen such a substrate, e.g., by preloading it with an overload embankment [12]. This type of soil improvement improves peat properties such as compressibility, stiffness, or permeability [2]. In that method, it is important to check the settlement of embankment for all times of consolidation. Buildings can be placed on the site after the consolidation is complete, which is why preloading of embankment is also a method to know the real value of the constrained modulus of elasticity. With these values, engineers can predict settlements closer to real settlements on these sites.

Research on the deformation of organic soils loaded with experimental embankments show large vertical and horizontal displacements in the zone of the loaded subsoil. Based on the observations, it can be seen that a large part of the horizontal deformation occurs during loading and shortly after its completion. Horizontal displacements do not play a significant role in the process of organic soil consolidation, except in the early stages of construction [7,22]. On this basis, calculations of the course of settlements and dispersion of the excess pore pressure in the soil under the embankment can be carried out per the one-dimensional consolidation theory, considering the appearance of plastic strains when loading the substrate [7,19,22].

The constrained modulus of elasticity *E* is poorly described in the literature, and the intention is to focus on its determination in the oedometric study described previously. However, the settlement of the embankment on organic soils has been described earlier, based on the cases of embankments on organic soils at research sites in Mielimaka, Białośliwie, and Antoniny (Poland, Central Europe) [7,19,22]. Test loads with embankments of organic soils were described by Huat, Ibrahim and his team, and Indraratna [2,23,24]. An interesting approach to the load on organic soils was demonstrated by the team of Waruwu and Rifa, who loaded the peat material with an embankment under laboratory conditions [25,26,27]. In these works, a constant constrained modulus of elasticity *E* was adopted, focusing on the consolidation of organic soils. The authors used a new approach to determine this peat parameter based on the presented models. Taking into account changes in vertical stresses in the soil and dimensions of the loaded area, designers can use the new approach to determine the modulus, while strengthening it. When determining the peat compressibility parameter, the authors analysed the relationship between the material and the load in conditions where there is no longer excess pore pressure and consolidation is complete.

This paper presents a method for the practical determination of the compressibility modulus of an organic soil layer in natural conditions, which will refine the calculation of settlement in engineering practice. The research was based on the total settlement of the embankment, i.e., after its consolidation.

The authors present the experimental studies performed on the embankment settlement on a natural scale. The oedometric modulus of organic soil compressibility to those obtained from research on-site was compared. The authors have created an original method for the practical description of changes in the constrained modulus of elasticity *E* of organic soils preloaded with an embankment, based on experimental research in the field. This method may eliminate the differences between the analytical calculations of soil settlement and the actual conditions found in the field.

## 2. Materials and Methods

The authors presented two methods to determine the constrained modulus of elasticity of peat material. In each method, the organic soil layer should be loaded with an overloading embankment, and its final settlement should be determined. The first method is based on the constant stress distribution as in the oedometer test. The second method includes the influence of vertical stresses from the entire surface of the embankment on the analysed place.

**Figure 2:**
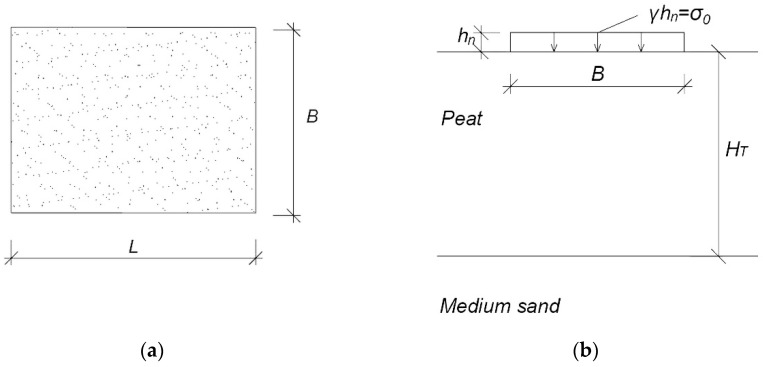
Scheme of the embankment: (**a**) top view; (**b**) cross-section. *B*, embankment width (m); *L*, embankment length (m); *h_n_*, height of the embankment (m); *γ*, weight of the embankment (kN m^−3^); *σ*_0_, loaded with the embankment (kPa); *H_T_*, thickness of organic soil (m).

The uniform loading of the organic soil with a preloaded embankment with dimensions *B* × *L* was used to determine the constrained modulus of elasticity, shown in Figure 2. The experiment does not include the inclination of slopes because their effect by such an extensive area of the embankment is not significant [6,28,29]. 

**Figure 3:**
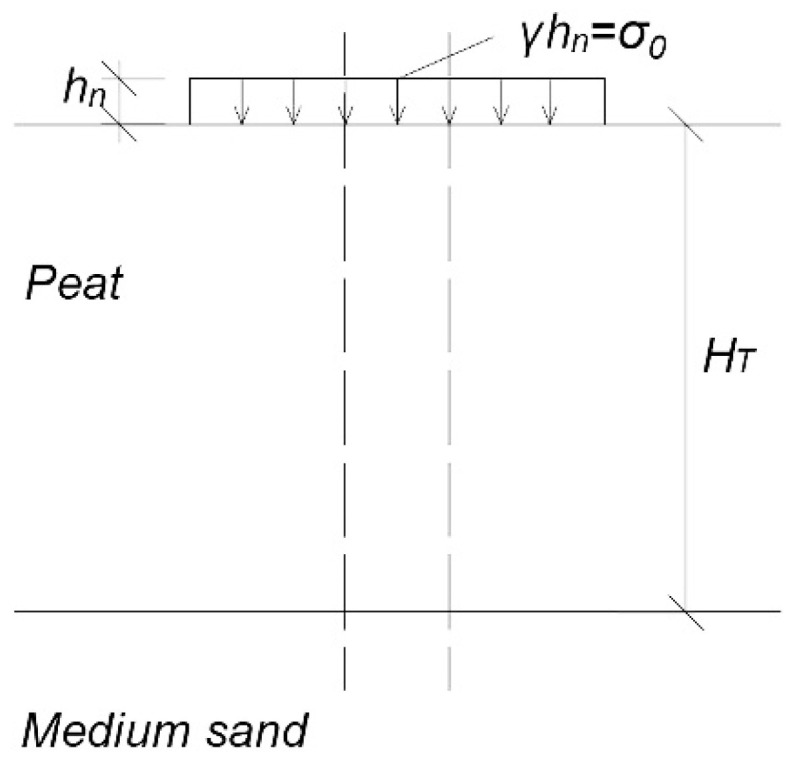
Diagram of the organic soil (peat) column in a separate calculation area; *h_n_*, height of the embankment (m); *γ*, weight of the embankment (kN m^−3^); *σ*_0_, loaded with the embankment (kPa); *H_T_*, thickness of the organic soil (m).

In the analysis of the stress–strain relationship, the embankment was divided into smaller rectangular calculation areas. Under each of them, there is a column of organic soil (peat) loaded from the top with an embankment layer, and the bottom is limited by a non-deformed compacted medium sand (Figure 3). 

Compression of the organic soil layer (peat material layer) by the embankment can be made of non-cohesive soil, e.g., sand. The body of the embankment can be built of fine, medium, coarse sands or coarse-grained compacted soils. One should strive to obtain soil moisture close to the optimal value, which will enable the achievement of the maximum volume density of the soil skeleton [6,12,30].

The layer of organic soil (peat) of known thickness *H_T_* has the properties described earlier, systematized in field and laboratory tests. The underlying layer can be, for example, sand, which is not compressible. The load applied to the peat layer is assumed to be value *σ_0_*, and the resulting stresses act on the calculation column as a vertical force. As a result, the column is compressed. In the paper, the authors assume that this compression is determined by a representative modulus for uniaxial compression [6,29].

**Figure 4:**
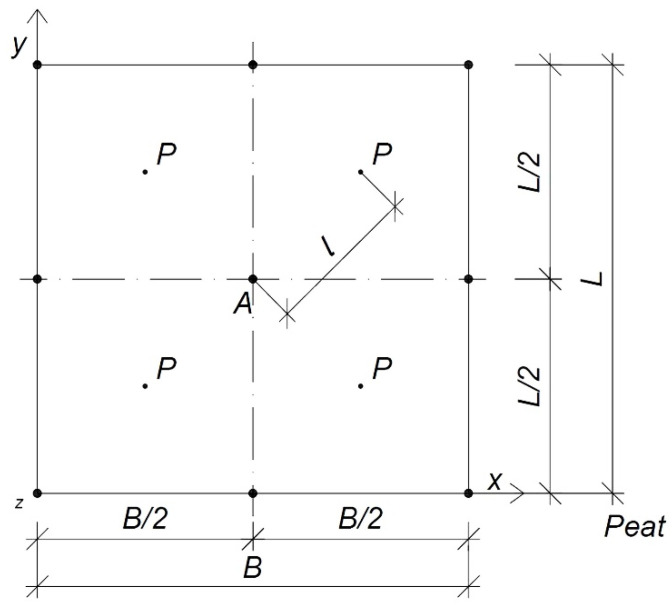
The division of the embankment into calculation areas for integration; top view; *P*, force (kN); *l*, distance from an analysed point to force (kN); *A*, analysed point (−); *B*, width of the embankment (m); *L*, length of the embankment (m); *x*, axis of width; *y*, axis of depth; *z*, axis of depth.

Calculations should be performed at points resulting from the division of the embankment. The force concentrated in its centre can be determined, as shown in Figure 4. According to the principle of superposition, one analysed place will be influenced by all other calculation fields [6,12,31]. 

When an embankment acts on the subsoil, each soil element (peat material) under the embankment at any depth is bounded by adjacent elements. These elements are subjected to a state of stress [32,33]. It was assumed that there are no horizontal deformations of the soil outside the boundaries of the loaded area. Compared to the thickness of the compressible soil under the embankments, there is no ideal one-dimensional compression condition. On-site, there is some settlement caused by lateral displacement of the soil from under the load area. However, inclinometer measurements of lateral soil deformation at the embankment boundaries indicate that settlements due to lateral deformations are generally small compared to compression settlements [32]. Therefore, the general method of calculating the settlement is based on the uniaxial state of strain. Consolidation is not a point of this paper, and the value of settlement is taken after the consolidation.

The embankment layer settled most in the middle and the least at the corners. Stresses from external load in the soil depend on its value, place of its application, and the area of action [6,31]. The stress is determined based on the Boussinesq theory and the principle of superposition [6,12,31], according to Equation (2):

(2) $$\sigma_{z}=\frac{3}{2\pi }\frac{Pz^{3}}{{\left(l^{2}+z^{2}\right)}^{\frac{5}{2}}}$$

where 

*P*—force acting on the subsoil in the analysed calculation area (kN); 

*z*—depth consideration space (m); 

*l*—distance of the force from the analysed point in the embankment determined according to Equation (3) (m):

(3) $$l=\sqrt{{\left(x_{0}-x\right)}^{2}+{\left(y_{0}-y\right)}^{2}}$$

where 

*x*, *y*—coordinates of the force applied in the embankment plane (m).

**Figure 5:**
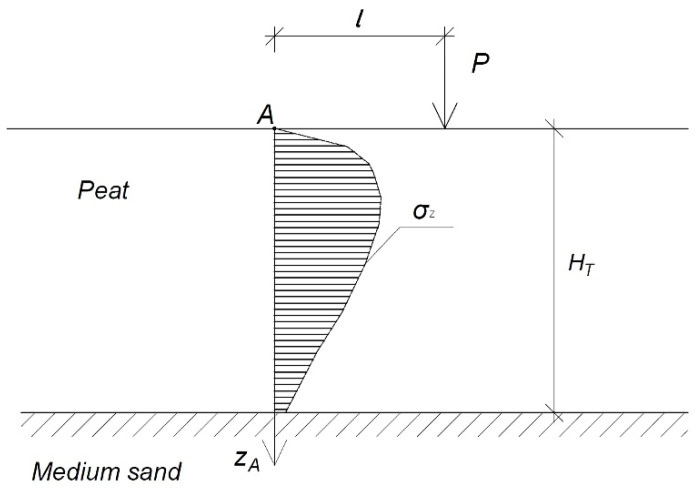
Stress distribution at distance *l* from the force *P* according to Boussinesq; *A*, analysed point (−); *P*, force (kN); *l*, distance from an analysed point to force (kN); *H_T_*–thickness of the organic soil (m); *σ_z_*, stress distribution in the soil (kPa); *z_A_*, axis of depth.

The diagram of the stress distribution in the centre according to the Boussinesq theory is shown in Figure 5 [6,29,31].

Stresses in the column at any depth “*z*” and the effect of load on the state of vertical stresses in the remaining columns can be written by Equation (4):

(4) $$\sigma_{z}=\frac{3}{2\pi }\int^{}\frac{\sigma_{0}dA}{{\left(z^{2}+l^{2}\right)}^{\frac{5}{2}}}$$

**Figure 6:**
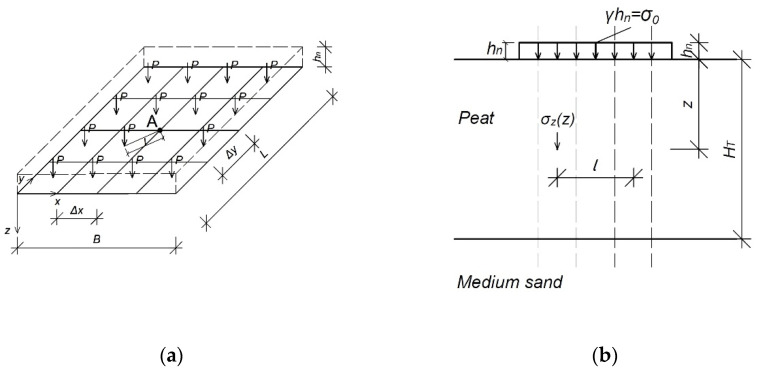
Influence of the calculation areas on each other: (**a**) division of the embankment into calculation areas and distance of the force to the point; (**b**) cross-section. *P*, force (kN); *l*, distance from an analysed point to force (kN); *A*, analysed point (−); *B*, width of the embankment (m); *L*, length of the embankment (m); *x*, axis of width; *y*, axis of depth; *z*, axis of depth; *Δx*, width of the mesh element; *Δy*, length of the mesh element; *h_n_*, height of the embankment (m); *γ*, weight of the embankment (kN m^−3^); *σ*_0_, loaded with the embankment (kPa); *H_T_*, thickness of the organic soil (m); *σ_z_*, stresses in the soil (kPa).

According to the superposition principle, in the analysed column of organic soil, the stresses will consider the influence of the load exerted on the analysed area and all the others (Figure 6) [6,29,31]. 

When analysing the selected calculation area/column, it is reasonable to use a uniaxial model that shows the stresses from other calculation fields. Only vertical stresses in the soil will be used to perform the calculations, and the analysed settlements will be complete, i.e., after the subsoil consolidation process is completed.

The constrained modulus of elasticity *E* can be determined by a model that is based on the constant stress distribution, as in the oedometer test (first model), and by reconciling the influence of the entire load area and the thickness of the organic soil under it (second model). Based on this, a map of the constrained modulus of elasticity variability under the preload embankment can be created. The least-squares method should approximate its value based on the values at the selected points to obtain the value of the constrained modulus of elasticity for the entire layer. This value of the constrained modulus of elasticity *E* can determine peat material settlement at the design stage.

The formulae for the constrained modulus of elasticity were derived, which in practical conditions, allows the use of a uniaxial deformation state with the combination of the total settlement. The authors describe all load and deformation components and transposed them into a uniaxial state of deformation, which corresponds to total settlement. Based on this, a calculation program was developed to determine the settlement–stress (load) relationship.

### 2.1. The First Model to Determine the Constrained Modulus of Elasticity in Peat Material

In the first model, calculations are made at selected points based on the discretization of the embankment. 

When using this model, it should be assumed that the external load stresses in the peat layer are equal *σ_z_* = *σ*_0_ = const. The influence of the load from other design areas is not taken into account. 

**Figure 7:**
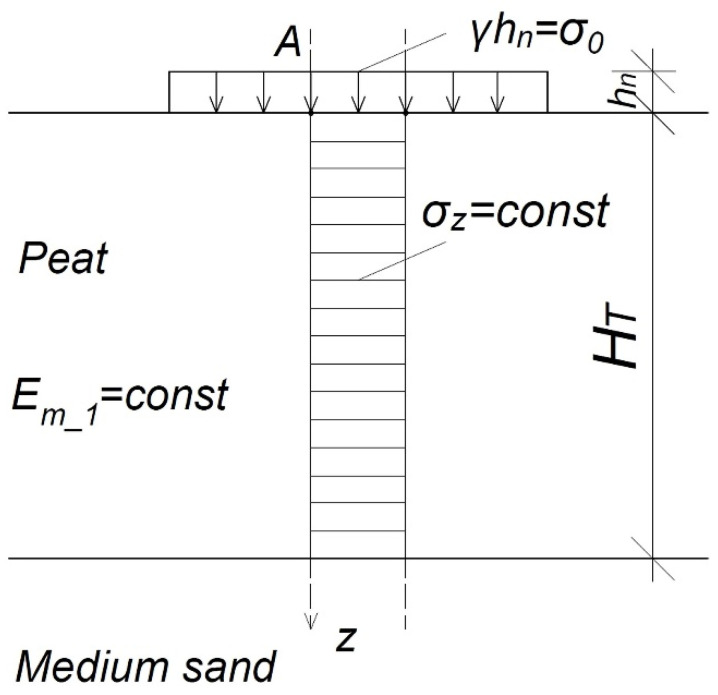
Computational organic soil column; *σ_0_*, loading by the preload embankment (kPa); *σ_z_*, stresses in the soil in the column under consideration (kPa); *H_T_*, organic soil thickness (m); *E_m_1_*, constrained modulus of elasticity of peat material in the column under consideration; determined by the first model (kPa); *A*, analysed point (−); *h_n_*, the height of the embankment (m); *γ*, the weight of the embankment (kN m^−3^); *z*, axis of depth.

Organic soil constrained modulus of elasticity (*E_m_1_*) is constant throughout the column (*E_m_1_* = const and $\frac{\partial E}{\partial z}=0$). The assumptions for the calculations are shown in Figure 7.

The calculated settlement of the peat material layer in Model 1 was defined as:

(5) $$s=\frac{\sigma_{0}}{E_{m\_1}}H_{T}$$

where

*s* —settlement of the organic soil layer (m);

*σ_0_* —load caused by the preload embankment (kPa);

*H_T_* —thickness of the organic soil layer (m);

*E_m_1_* —constrained modulus of elasticity in the considered column determined by the first model (kPa).

Knowing the settlement in the analysed place of the embankment and using the above dependence, it is possible to determine the organic soil constrained modulus of elasticity by the first model with Equation (6).

(6) $$E_{m\_1}=\frac{\sigma_{0}}{s}H_{T}$$

### 2.2. The Second Model to Determine the Constrained Modulus of Elasticity in Peat Material

The second model contains information about the influence of load acting at any point in the load area of the column and the formation of vertical stress in the soil. The calculations are performed in points determined by the division of the embankment into calculation areas.

**Figure 8:**
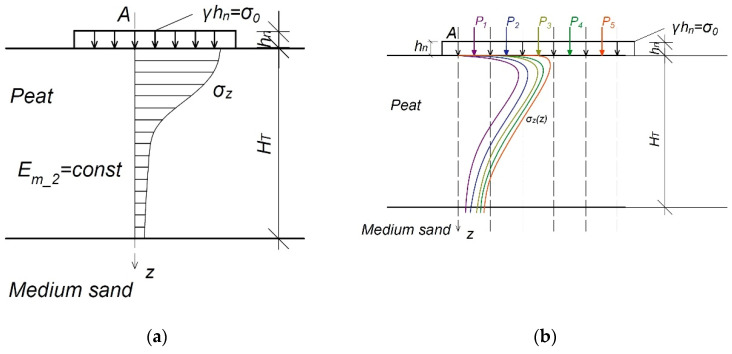
The scheme adopted in the second model: (**a**) calculating a column of soil organic; (**b**) influence of the load from the remaining calculation areas on the stress distribution in the analyzed point; *P_1–5_*, forces acting in the centre of the calculation area (kN); *A*, analysed point (−); *h_n_*, the height of the embankment (m); *γ*, the weight of the embankment (kN m^−3^); *z*, axis of depth.

The Boussinesq theory and the principle of superposition are applied. The distribution in the organic soil layer is not constant: *σz = f(x,y,z)*, $\frac{\partial \sigma_{z}}{\partial z}\neq 0$. In the second model, the influence of the remaining calculation areas is taken into account, following the principle of superposition (Figure 8b).

The compressibility modulus determined by the second model is constant throughout the peat layer: *E_m_2_* = const and in the column: $\frac{\partial E}{\partial z}=0$. 

The assumptions for the calculations are outlined in Figure 8a.

The second model is analysed at any point *A* with the coordinates (*x_A_*, *y_A_*), according to the adopted coordinate system (e.g., Figure 6), where $x_{A}\in \langle 0;B\rangle$ and $y_{A}\in \langle 0;L\rangle$. 

The stresses in the organic soil column will be calculated according to the Boussinesq theory:

(7) $$\sigma_{z}=\frac{3}{2\pi }\frac{Pz^{3}}{{\left(l^{2}+z^{2}\right)}^{\frac{5}{2}}}$$

where

*P*—force acting on the subsoil in the analysed calculation area [kN]; 

l—distance of the force from the analysed point in the embankment determined according to Equation (8) (m):

(8) $$l=\sqrt{{\left(x_{A}-x\right)}^{2}+{\left(y_{A}-y\right)}^{2}}$$

where

*x, y* —coordinates of the application of the force in the plane of the embankment (m);

*x_A_, y_A_* —coordinates of the analysed point (m).

The vertical force *P* on any elementary area of the embankment is determined by:

(9) $$P=\sigma_{0}dA=\sigma_{0}dxdy$$

where

*A* —calculated area field (m^2^);

*σ_0_* —embankment load (kPa).

The total calculation for settlement of the peat layer in the second model is presented as:

(10) $$s=\frac{\int_{0}^{B}\left(\int_{0}^{L}\left(\int_{0}^{H_{T}}\sigma_{z}dz\right)dy\right)dx}{E_{m\_2}}$$

Concerning the model presented in the earlier work [6], the authors consider the influence of embankment stresses at a given point by integrating the entire embankment area, not summing up the previously calculated stress values at each assumed point.

The stresses in the organic soil layer at a point are determined based on Equation (11) [6]:

(11) $$I_{i}=\int_{0}^{H_{T}}\sigma_{z}dz=\frac{3\sigma_{0}A}{2\pi }\left[\frac{2}{3l}-\frac{2l^{2}+3H_{T}{}^{2}}{3{\left(l^{2}+H_{T}{}^{2}\right)}^{3/2}}\right]$$

Taking into account the influence of the remaining calculation areas on point *A* and the change of stresses in the soil, it can be concluded that:

(12) $$\overset{B}{\underset{0}{\int }}\left(\overset{L}{\underset{0}{\int }}\left(\overset{H_{T}}{\underset{0}{\int }}\sigma_{z}dz\right)dy\right)dx=\int_{0}^{L}\int_{0}^{B}I_{i}dxdy=\int_{0}^{L}\int_{0}^{B}\left\{\frac{3\sigma_{0}A}{2\pi }\left[\frac{2}{3l}-\frac{2l^{2}+3H_{T}{}^{2}}{3{\left(l^{2}+H_{T}{}^{2}\right)}^{3/2}}\right]\right\}dxdy$$

Assuming $J_{i}=\int_{0}^{B}\left(\int_{0}^{L}\left(\int_{0}^{H_{T}}\sigma_{z}dz\right)dy\right)dx$, Equation (13) should be solved as follows:

(13) $$J_{i}=\overset{B}{\underset{0}{\int }}\left(\overset{L}{\underset{0}{\int }}\left(\overset{H_{T}}{\underset{0}{\int }}\sigma_{z}dz\right)dy\right)dx=\frac{\sigma_{0}}{2\pi }\left(A_{i}+B_{i}+C_{i}+D_{i}+E_{i}+F_{i}\right)$$

where parameters *A_i_*, *B_i_*, *C_i_*, *D_i_*, *E_i_*, *F_i_* are variable expressions determined by Equations (14) to (19):

(14) $$A_{i}=-H_{T}\left[\text{ArcTan}\left[\frac{x_{A}\left(L-y_{A}\right)}{H_{T}\sqrt{H_{T}{}^{2}+x_{A}{}^{2}+{\left(L-y_{A}\right)}^{2}}}\right]+\text{ArcTan}\left[\frac{x_{A}y_{A}}{H_{T}\sqrt{H_{T}{}^{2}+x_{A}{}^{2}+y_{A}{}^{2}}}\right]\right]$$

(15) $$B_{i}=-H_{T}\left[\text{ArcTan}\left[\frac{\left(B-x_{A}\right)\left(L-y_{A}\right)}{H_{T}\sqrt{H_{T}{}^{2}+{\left(B-x_{A}\right)}^{2}+{\left(L-y_{A}\right)}^{2}}}\right]+\text{ArcTan}\left[\frac{\left(B-x_{A}\right)y_{A}}{H_{T}\sqrt{H_{T}{}^{2}+{\left(B-x_{A}\right)}^{2}+y_{A}{}^{2}}}\right]\right]$$

(16) $$C_{i}=-2\left[-L+\left(L-y_{A}\right)\text{Log}\left[-x_{A}+\sqrt{x_{A}{}^{2}+{\left(L-y_{A}\right)}^{2}}\right]-x_{A}\text{Log}\left[L+\sqrt{x_{A}{}^{2}+{\left(L-y_{A}\right)}^{2}}-y_{A}\right]+y_{A}\text{Log}\left[-x_{A}+\sqrt{x_{A}{}^{2}+y_{A}{}^{2}}\right]+x_{A}\text{Log}\left[-y_{A}+\sqrt{x_{A}{}^{2}+y_{A}{}^{2}}\right]\right]$$

(17) $$D_{i}=2[-L+H_{T}\text{ArcTan}[\frac{L-Y_{A}}{H_{T}}]+H_{T}\text{ArcTan}\left[\frac{x_{A}\left(L-y_{A}\right)}{H_{T}\sqrt{H_{T}{}^{2}+{x_{A}}^{2}+{\left(L-y_{A}\right)}^{2}}}\right]+H_{T}\text{ArcTan}[\frac{y_{A}}{H_{T}}]\;+H_{T}\text{ArcTan}[\frac{x_{A}y_{A}}{H_{T}\sqrt{H_{T}{}^{2}+{x_{A}}^{2}+{y_{A}}^{2}}}]+y_{A}\text{Log}\left[-x_{A}+\sqrt{H_{T}{}^{2}+x_{A}{}^{2}+y_{A}{}^{2}}\right]\;+x_{A}\text{Log}\left[-y_{A}+\sqrt{H_{T}{}^{2}+x_{A}{}^{2}+y_{A}{}^{2}}\right]+L\text{Log}\left[-x_{A}+\sqrt{H_{T}{}^{2}+(L-y_{A}){}^{2}+x_{A}{}^{2}}\right]\;-y_{A}\text{Log}\left[-x_{A}+\sqrt{H_{T}{}^{2}+(L-y_{A}){}^{2}+x_{A}{}^{2}}\right]-x_{A}\text{Log}\left[L-y_{A}+\sqrt{H_{T}{}^{2}+(L-y_{A}){}^{2}+x_{A}{}^{2}}\right]$$

(18) $$E_{i}=2\left[-L+\left(L-y_{A}\right)\mathrm{Log}\left[B-x_{A}+\sqrt{{\left(B-x_{A}\right)}^{2}+{\left(L-y_{A}\right)}^{2}}\right]+\;\left(B-x_{A}\right)\mathrm{Log}\left[L+\sqrt{{\left(B-x_{A}\right)}^{2}+{\left(L-y_{A}\right)}^{2}}-y_{A}\right]+y_{A}\mathrm{Log}\left[B-x_{A}+\sqrt{{\left(B-x_{A}\right)}^{2}+y_{A}{}^{2}}\right]\;-\left(B-x_{A}\right)\mathrm{Log}\left[-y_{A}+\sqrt{{\left(B-x_{A}\right)}^{2}+y_{A}{}^{2}}\right]\right]$$

(19) $$F_{i}=2[L+H_{T}[-\text{ArcTan}[\frac{L-Y_{A}}{H_{T}}]+\text{ArcTan}\left[\frac{\left(B-x_{A}\right)\left(L-y_{A}\right)}{H_{T}\sqrt{H_{T}{}^{2}+{\left(B-x_{A}\right)}^{2}+{\left(L-y_{A}\right)}^{2}}}\right]-\text{ArcTan}[\frac{y_{A}}{H_{T}}]\;+\text{ArcTan}[\frac{(B-x_{A})y_{A}}{H_{T}\sqrt{H_{T}{}^{2}+{\left(B-x_{A}\right)}^{2}+{y_{A}}^{2}}}]]+\;(-L+y_{A})\text{Log}\left[B-x_{A}+\sqrt{H_{T}{}^{2}+(B-x_{A}){}^{2}+(L-y_{A}){}^{2}}\right]\;-y_{A}\text{Log}\left[B-x_{A}+\sqrt{H_{T}{}^{2}+(B-x_{A}){}^{2}+y_{A}{}^{2}}\right]\;-(B-x_{A})[\text{Log}[L+\sqrt{H_{T}{}^{2}+(B-x_{A}){}^{2}+(L-y_{A}){}^{2}}-y_{A}]\;-\text{Log}\left[-y_{A}+\sqrt{H_{T}{}^{2}+(B-x_{A}){}^{2}+y_{A}{}^{2}}\right]]]]$$

where

*x_A_*, *y_A_* —coordinates of the analysed point (m);

*B* —embankment width (m);

*L* —embankment length (m);

*H_T_* —thickness of the organic soil layer (m).

The total computational settlement of organic soil containing the relationship (10) according to the second model can be determined:

(20) $$s=\frac{J_{i}}{E_{m\_2}}=\frac{\sigma_{0}\left(A_{i}+B_{i}+C_{i}+D_{i}+E_{i}+F_{i}\right)}{2\pi E_{m\_2}}$$

Knowing the load on the embankment and the settlement of organic soil, the constrained modulus of elasticity according to the second model can be represented by Equation (21):

(21) $$E_{m\_2}=\frac{J_{i}}{s}=\frac{\sigma_{0}\left(A_{i}+B_{i}+C_{i}+D_{i}+E_{i}+F_{i}\right)}{2\pi \;s}$$

## 3. Results

The embankment erected on organic soils was numerically analysed. Then, the peat compressibility modulus was determined under natural conditions to verify the proprietary solution.

### 3.1. Numerical Simulations for the First and Second Method

**Figure 9:**
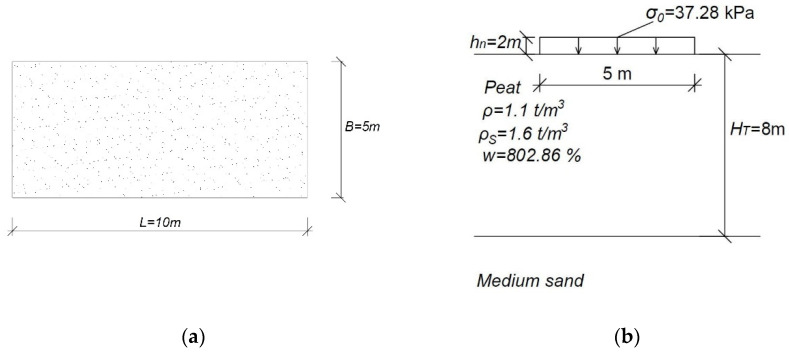
Conditions adopted for the analyses: (**a**) dimensions of the embankment; (**b**) cross-section.

It was assumed that the dimensions of the embankment were *B* = 5 m, *L* = 10 m, and height *h_n_* = 2 m. The embankment was made of the compacted, moist medium sand with *γ_embankment_*=18.64 kN m^−3^. The load exerted on the subsoil was *σ*_0_ = 37.28 kPa. The embankment was founded on a layer of peat material with a thickness of *H_T_* = 8 m. Basic parameters of the peat material under the embankment were assumed: *ρ* = 1.1 t/m^3^, *ρ_s_* = 1.6 t/m^3^ and moisture 802.86%. The above conditions are shown in Figure 9.

**Figure 10:**
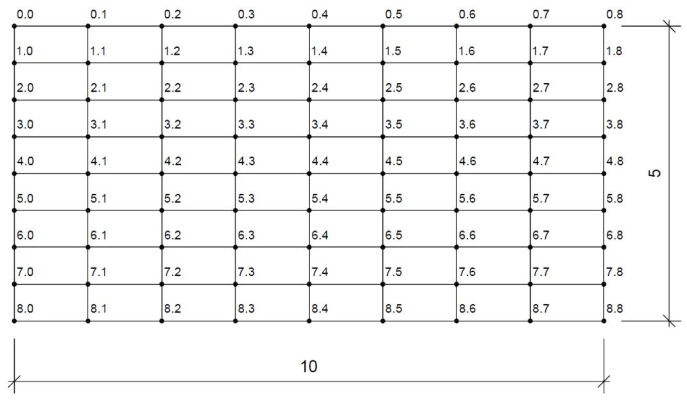
Division of the adopted embankment into calculation areas—numbering of calculation points.

The embankment was divided into smaller calculation areas. Points and forces acting inside each field were determined with the use of a proprietary calculation program. The division of the embankment is shown in Figure 10.

It was assumed that the total settlement (*s_t_*) of the embankment was most significant in the middle and most minor in the corners. Its maximum value is 0.48 m and the minimum at 0.12 m. Final settlement values are shown in Table 1.

#### 3.1.1. Determining the Constrained Modulus of Elasticity with the First Model in Peat Material for Numerical Simulation

**Figure 11:**
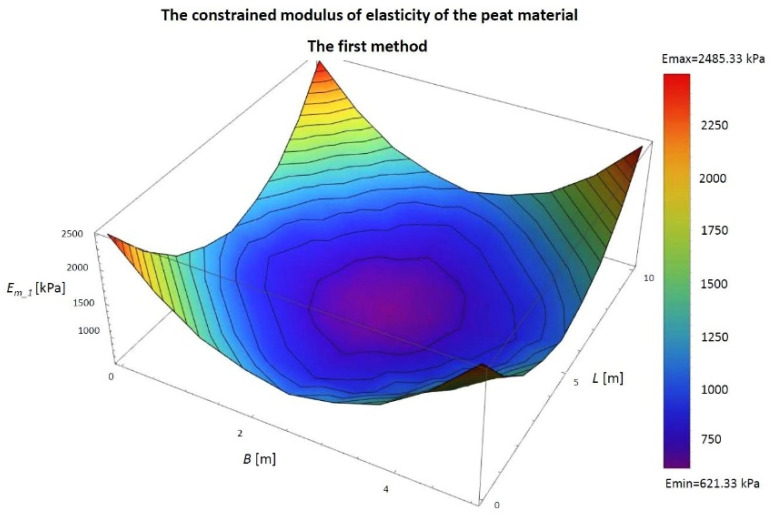
The first model determines the constrained modulus of elasticity of the peat material. *B*, width of the embankment (m); *L*, length of the embankment (m); *E_m_1_*, constrained modulus of elasticity of peat material in the column under consideration, determined by the first model (kPa).

For the case described above, the constrained modulus of elasticity of the peat materials at the designated points was determined, as described in the first method. The constrained moduli of elasticity of peat loaded with a 5 × 10 m embankment are included in Table 2 and are shown in Figure 11.

From the obtained results, the largest constrained modulus of elasticity *E_m_1_* is at the corners at 2485.33 kPa, and the smallest was at the centre of the embankment at 621.33 kPa. The average is 1164.27 kPa (Figure 11 and Table 2). The difference between the maximum and minimum value is 1864.00 kPa.

The shape of the diagram shows the inverse variability to the settlement diagram. The lowest settlement occurred where the module is most significant, and the greatest is the lowest. This opposite is the result of the assumptions used in the first model.

#### 3.1.2. Determining the Constrained Modulus of Elasticity with the Second Model in Peat Material for Numerical Simulation

**Figure 12:**
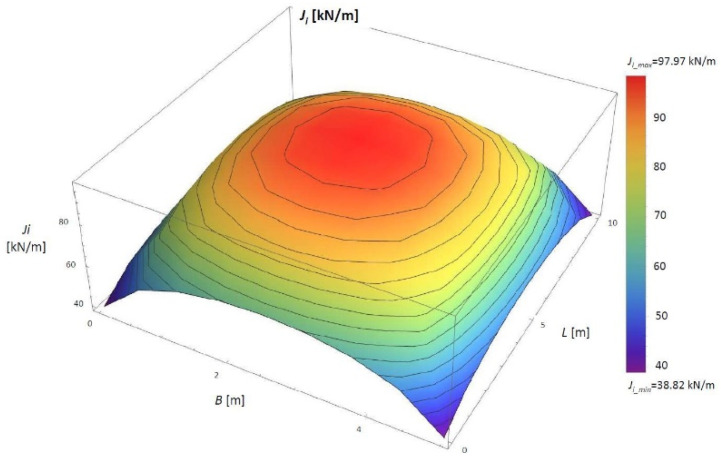
The values of the *J_i_* parameter in the 3D view for the numerical simulation. *B*, width of the embankment (m); *L*, length of the embankment (m).

The numerical analysis was performed for the assumed case. Then, the constrained modulus of elasticity of the peat material was determined using the second method. A parameter *J_i_* (kN/m) was first established to apply this method. The values of the *J_i_* (kN/m) parameter can be found in Figure 12 and Table 3.

The solution presented in Figure 12 shows that the most significant values of Ji are below the embankment’s centre (*J_i_max_* = 97.97 kN/m), and the smallest are at the corners (*J_i_min_* = 38.82 kN/m). The distribution of *Ji* confirms the correctness of these findings.

**Figure 13:**
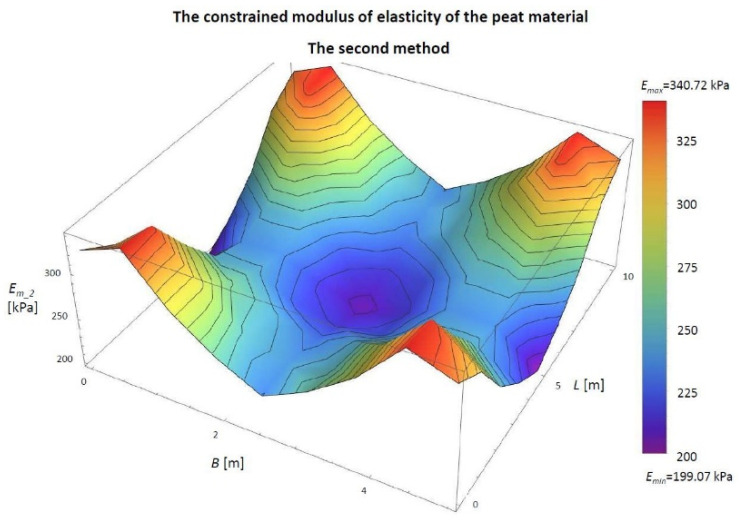
The values of the *J_i_* parameter in the 3D view for the numerical simulation. The constrained modulus of elasticity of peat material determined by the second model (*E_m_2_*) in the 3D view. *B*, width of the embankment (m); *L*, length of the embankment (m).

Then, the constrained modulus of elasticity value was determined using the Ji parameter and the final settlement value at individual points. The results are shown in Table 4 and Figure 13.

Figure 13 shows that the largest modules *E_m_2_* are close to the corners (points: 1.0, 1.8, 7.0, 7.8), and their value is 340.72 kPa. The smallest values are in the middle (point: 4.4) *E_m_2_* = 204.11 kPa and in the middle parts of the embankment length (points: 0.4 and 8.4) *E_m_2_* = 199.07 kPa. The difference between the maximum and minimum value of the module is 141.65 kPa.

### 3.2. Full-Scale Experiment

After checking the models during numerical simulations, a full-scale experiment was performed.

The experimental tests were carried out at the test site on the Ostrów Brdowski Island in Szczecin, at the construction site of a factory for windmills. An experimental plot with a preload embankment was used. The embankments were founded on a layer of low peat, under which there are sands of valley bottoms and floodplain terraces. The peat material thickness is 9 m [6,31,34].

**Figure 14:**
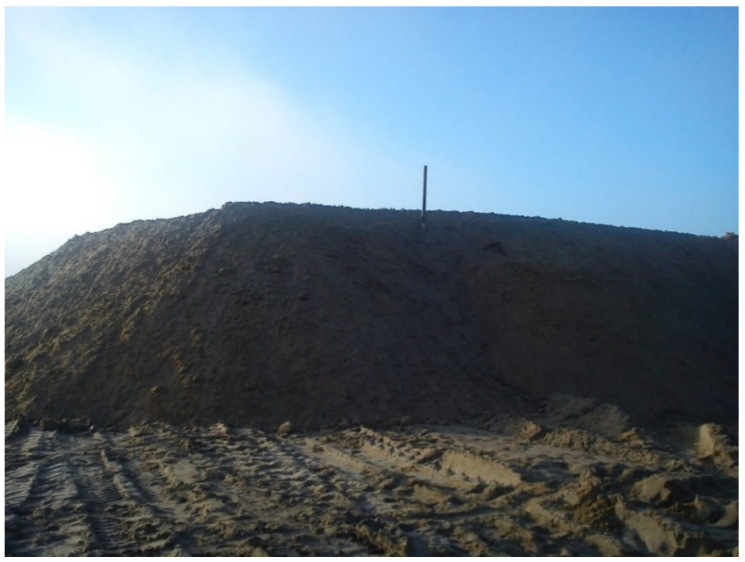
The preloading embankment at Ostrów Brdowski in Szczecin with a disc benchmark visible (photo M. Olszewska).

The subject of the experiment was the settlement process of the preload embankment. The data on the stress–strain relationship of peat material was obtained by reading the settlement of the disc benchmarks at fixed surface points at specific measurement points. The embankment is shown in the photo (Figure 14).

The calculations took into account the variability of the constrained modulus of elasticity and vertical stress. The result obtained in this way reflects the features found in natural conditions. Organic soil samples were taken to determine geotechnical parameters. For comparison, the authors also performed traditional tests using an oedometer.

**Figure 15:**
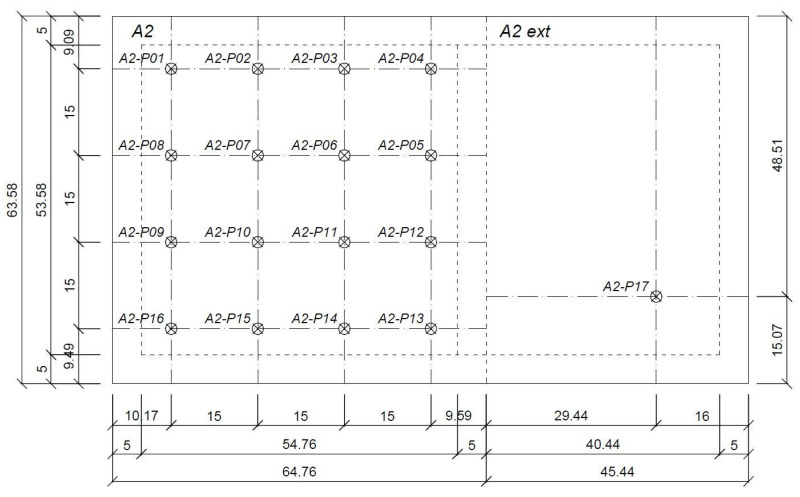
The embankment with marked settlement in measurement points from A2-P01 to A2-P17.

The embankment size is *B* = 63.58 m, *L* = 110.2 m, and it has two sections: A2, which contains measuring points from A2-P01 to A2-P16, and A2 ext, with the A2-P17 measuring point. The scheme of the embankment with the locations of the measurement points is shown in Figure 15.

**Figure 16:**
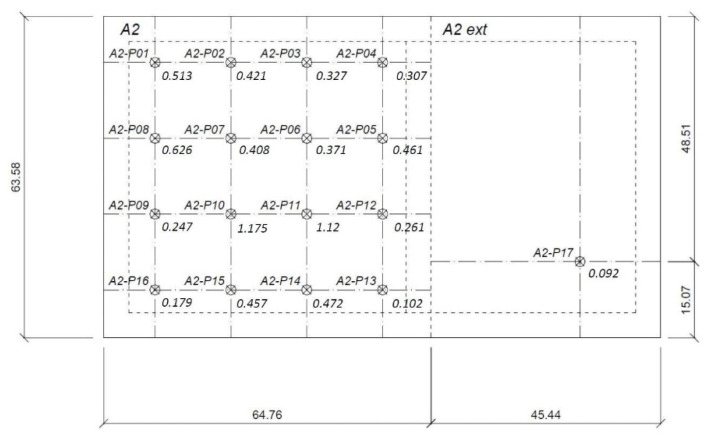
The embankment with marked settlement in measurement points from A2-P01 to A2-P17.

The embankment loaded the subsoil. The load value was equal to σ_0_ = 35 kPa. The embankments were observed for 84 days. A vertical drainage system was used to complete the total settlement faster. The values of total settlement at the indicated points are summarized in Table 5. The total settlement of the embankment is shown in Figure 16. 

In the embankment, the highest settlement value reached 1.175 m at the A2-P10 point and reached the lowest of 0.092 m at A2-P17, with a mean value of 0.410 m. 

The authors used the above values to determine the constrained modulus of elasticity of the peat material layer based on its overload with an embankment by the described methods. 

One of the essential elements when changing the volume under load is rheological properties. They make it possible to consider changes in the behaviour of the sample, even if they have only structural changes in a fragmented medium [35]. An example of this is self-propelled colloidal populations that induce time-dependent three-dimensional fluid flows, which change the rheological properties of the centres [35]. The authors did not consider this issue in this paper.

#### 3.2.1. Determining the Constrained Modulus of Elasticity with the First Model in Peat Material for a Full-Scale Experiment

Based on the previously described method, the constrained modulus of elasticity was determined for the presented case of a full-scale experiment.

**Figure 17:**
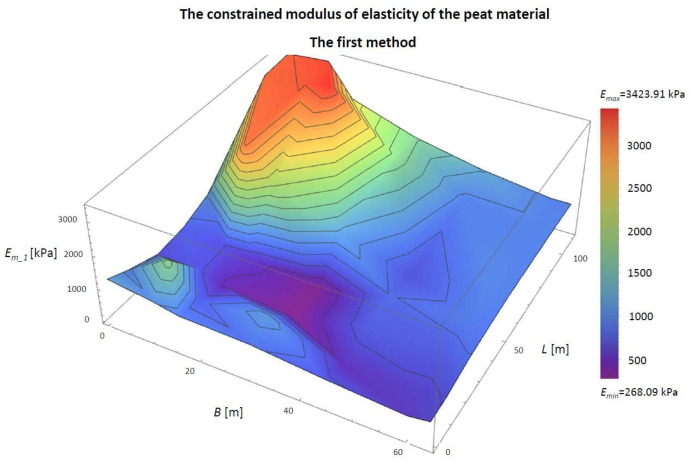
Distribution of the constrained modulus of elasticity values for the peat material determined with the first model under the embankment in Szczecin.

The constrained modulus results for the actual appendage in Szczecin are summarized in Table 6 and Figure 17.

For the full-scale experiment, the largest modulus is at the A2-P17 point and amounts to 3423.91 kPa, while the smallest is at the A2-P10 point (*E_m_1_* = 268.09 kPa). The average of all points is 1107.02 kPa. 

#### 3.2.2. Determining the Constrained Modulus of Elasticity with the Second Model in Peat Material for a Full-Scale Experiment

**Figure 18:**
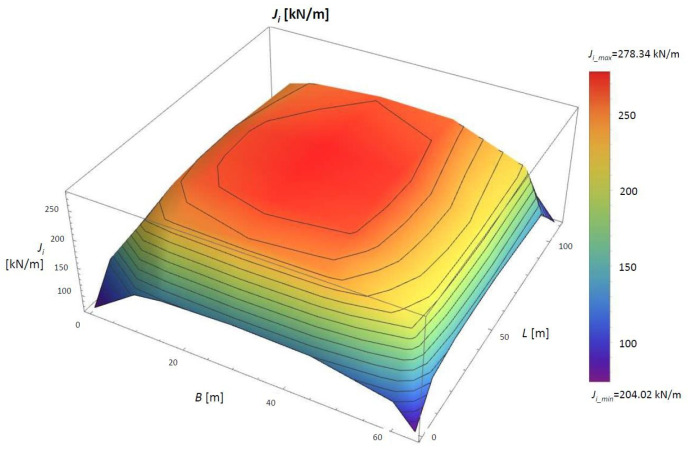
The values of the *J_i_* parameter for the embankment in Szczecin in the 3D view.

For the case described above and following the previously adopted assumptions, the *J_i_* parameter was calculated in the calculation points of the embankment. The result is shown in Table 7 and Figure 18.

Figure 18 and Table 7 show that the highest value *J_i_*, *J_i_max_* = 278.34 kN/m, is located at A2-P12 below the centre of the embankment, and the smallest, *J_i_min_* = 204.01 kN/m, in A2-P01 (*J_i_min_* = 204.01 kN/m). The span between these results is 74.32 kN/m.

**Figure 19:**
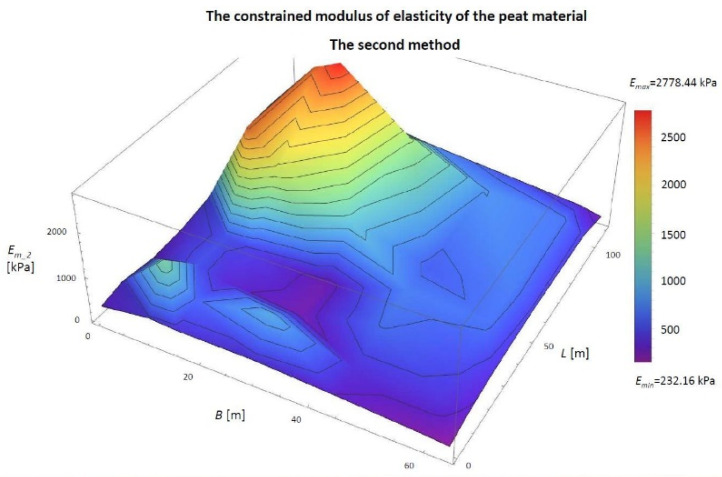
The second method is the value of the constrained modulus of elasticity under the embankment in Szczecin on the Ostrów Brdowski island.

Based on the known total settlement of the embankment at the points and the value of the *J_i_* parameter, the constrained modulus of elasticity of the peat material was determined using the second method. The results are shown in Table 8 and Figure 19.

According to Figure 19, the largest modulus, *E_m_2_* = 2743.17 kPa, appears at the point A2-P17, the place of the lowest embankment settlement. The smallest, *E_m_2_* = 232.16 kPa, in A2-P10, where settlement is the most intense. The mean value obtained from the calculations presented above is 877.62 kPa

## 4. Discussion

The constrained modulus of elasticity of the peat material was determined via both methods presented for the numerical simulation and the actual case study in Szczecin.

**Figure 20:**
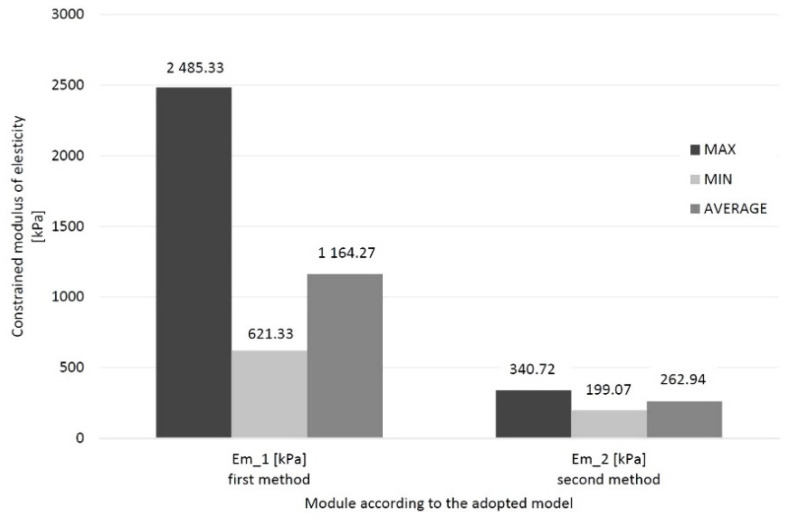
Two models determined the minimum, maximum, average and median values of the peat material constrained modulus of elasticity.

In numerical simulations, the constrained modulus of elasticity was obtained from the first model from 621.33 to 2485.33 kPa, and in the second model from 199.07 to 340.72 kPa. Their minimum, maximum and average values are presented in the diagram below (Figure 20).

For the simulation, the span between the maximum and minimum values for the first method is 1864 kPa, and the second is 141.65 kPa. It may prove that the influence of the loaded surface is of great importance. It is the result of made assumptions. The modulus is 4.43 times greater on the mean of the first method than on the second.

**Figure 21:**
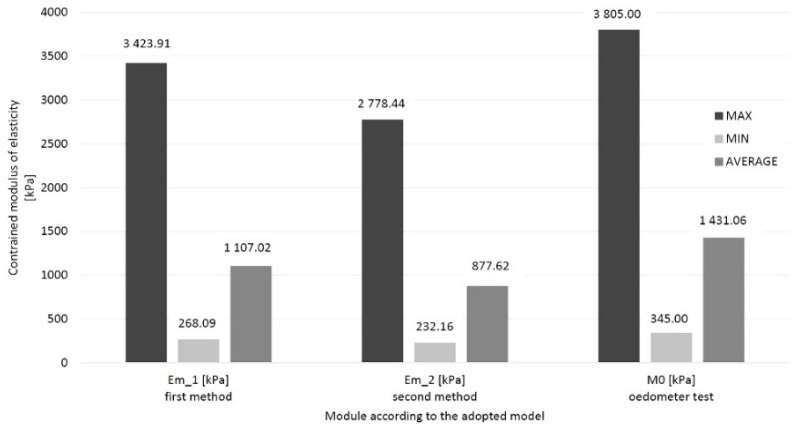
The minimum, maximum and average values for both methods and the results of the oedometric tests for the full-scale experiment on the Brdowski Island in Szczecin (Poland).

An embankment with B = 63.58 m, L = 110.2 m, and with 17 measuring points was made. The settlement was measured at all these points until the process stabilized and consolidation was completed. The total settlement was the basis for selecting the peat-constrained modulus of elasticity, which will consider the triaxial state of stresses and the uniaxial state of strains. Figure 21 presents the minimum, maximum and average values for both methods and the results of the oedometric tests for the full-scale experiment on the Brdowski Island in Szczecin (Poland).

For a full-scale experimental test, the spread between the maximum and minimum value is, respectively, 3155.83 kPa, and the second method is 2546.28 kPa. The constrained modulus of elasticity is 1.26 times greater on the mean of the first method than on the second. The average modulus tested in the oedometer is bigger than both the first (29% greater) and the second (63% greater).

**Figure 22:**
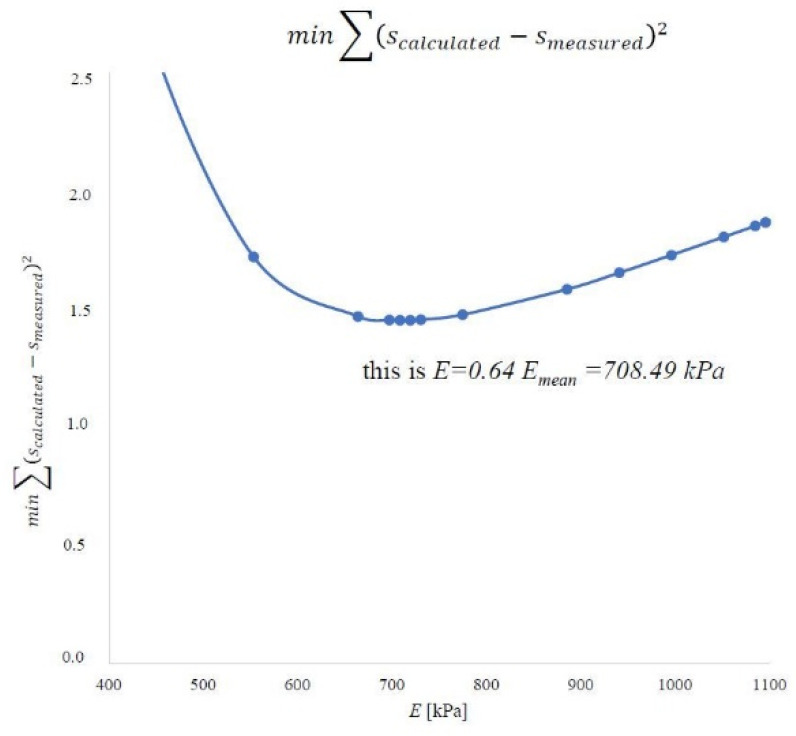
According to the first method, the representative value of the constrained modulus of elasticity of the peat material on the island of Ostrów Brdowski in Szczecin.

**Figure 23:**
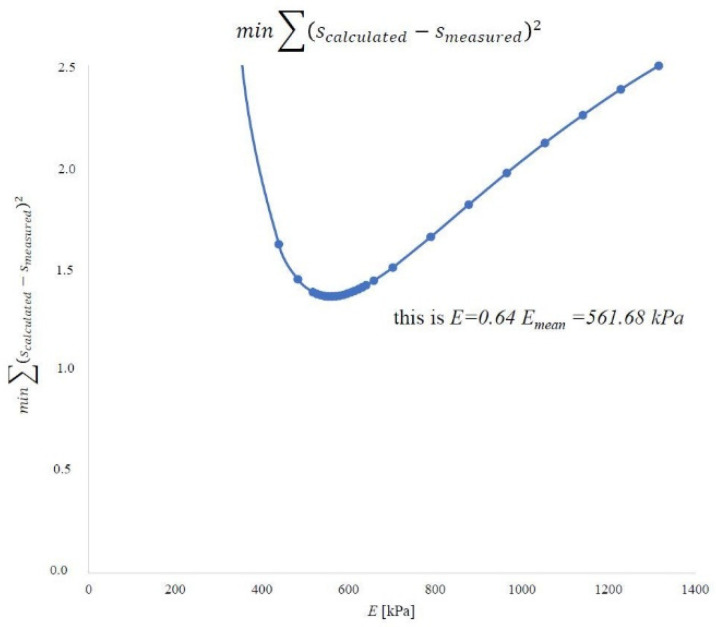
According to the second method, the representative value of the constrained modulus of elasticity of the peat material on the island of Ostrów Brdowski in Szczecin.

**Figure 24:**
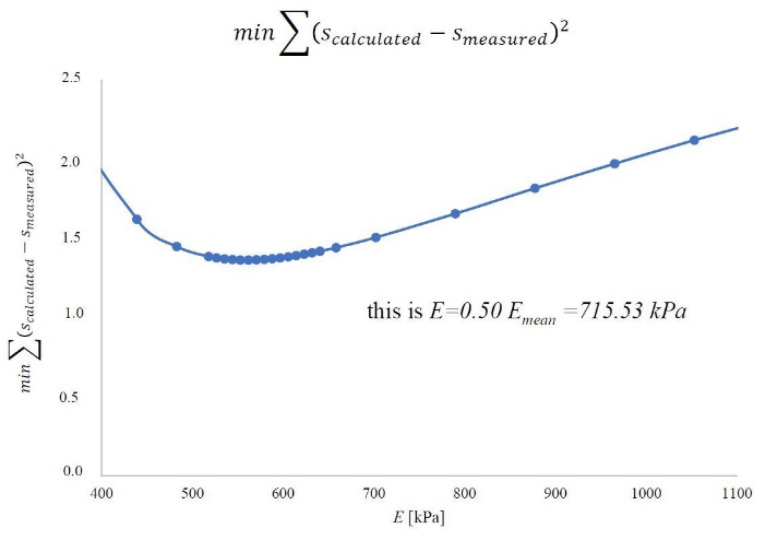
The representative value of the constrained modulus of elasticity of the peat material on the Ostrów Brdowski island in Szczecin according to the oedometer test.

By performing experimental tests and analyses, we know the dimensions of the embankment (*B* = 63.58 m and *L* = 110.2 m) and its height (*h_n_* = 1.9 m). The unit weight of the embankment material was 18.42 kN m^−3^ based on the research. The load on the peat was 35 kPa. The final settlement was known in the specified ones after completing the consolidation process. Subsequently, the strictness modules were determined by the methods described. On this basis, the constrained modulus of elasticity in the peat layer was optimized using the least-squares method. In engineering practice, those representative values of the constrained modulus of elasticity are determined for the entire soil layer to determine the predicted settlement of the object. For this purpose, the optimized value of the peat material constrained modulus of elasticity was determined using the least-squares method, referring to the calculated value and the measured settlement. The calculated settlement from each point was compared to the measurements. The results are shown for the first method in Figure 22, the second method in Figure 23, and the oedometer test in Figure 24.

**Figure 25:**
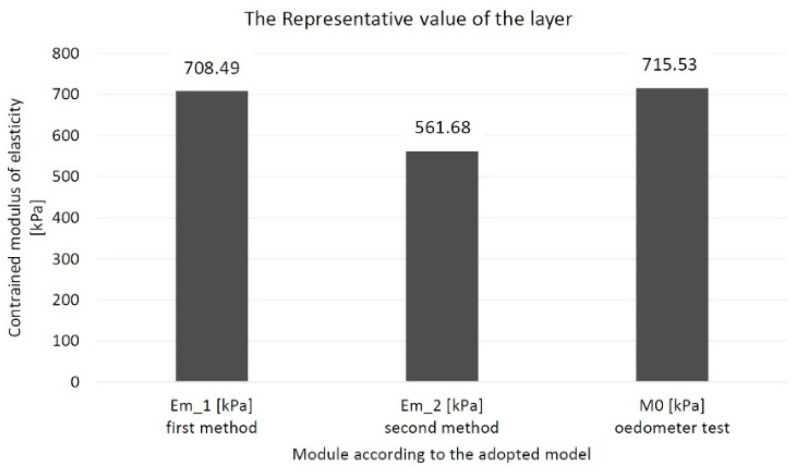
The representative values of the constrained modulus of elasticity for the peat layer according to the method used.

For the first method, the representative value is 708.49 kPa and 64% of the average value. In the second method, this value is 561.68 kPa and constitutes 64% of the average value. In an oedometer test, the representative value is 715.33 kPa and is 50% of the mean value. A summary of these values is shown in Figure 25.

The first method is based on the constant stress distribution. It was based on the oedometric model of the consolidation description relationship, assuming the effects of soil deformation and vertical filtration in the column [32]. In this method, the stress distribution is constant for the entire layer, *σ_z_* = const., in the peat column. Due to the constant stress distribution, the constrained modulus of elasticity results can be compared to those obtained in the oedometer. It can be seen in Figure 25 where the value of the representative constrained modulus of elasticity for the layer of peat material considered in the first method in the oedometer test has a similar value.

The second method makes the expected results more likely. Its determinants are: defining the compressibility modulus as a constant value over the entire thickness of the organic soil column, considering the variable vertical stress in the peat, and the influence of the load on the entire embankment. It is in line with Boussinesq’ s theory and is used for the practical calculation of settlement. In the case of peat material, this is of great importance. The second method considers more details than the first.

**Figure 26:**
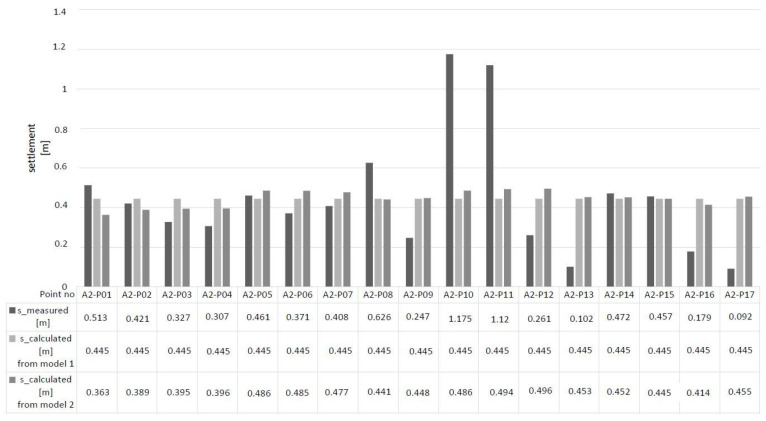
The representative values of the constrained modulus of elasticity for the peat layer, according to the method used.

For the optimized tightness modulus determined by Models 1 and 2, the settlement was calculated and then compared with the actual settlement of the embankment at each measured point (from A2-P01 to A2-P17). The comparison is shown in Figure 26.

From Figure 26, it can be seen that there are differences between the measured settlement and that calculated from the optimized value of modulus. One of the significant differences is that the settlement calculated from the compressibility modulus of Model 1 is a constant value at each point; however, this is not so. Model 1 assumes constant stresses in the soil, which allows the value to be related to the oedometer conditions. These conditions have an impact on the calculated settlement value. Nevertheless, the most similar results were obtained in points A2-P02, A2-P05, A2-P07, A2-P14 and A2-P15. The pair of points A2-P02 and A2-P07, as well as A2-P14 and A2-P15, are located next to each other. The greatest plains are at points A2-P10 and A2-P11 under the centre of the embankment. In Model 2, the stress values in the peat layer varied, and the influence of the entire loaded area was taken into account. Adding only this effect made the calculated settlement values variable, bringing the results closer to the real case. In Model 2, the stress values in the peat layer varied, and the influence of the entire loaded area was taken into account. Adding only this effect made the calculated settlement values variable. This value brings the results closer to the real case. Similar settlement results are in points A2-P14 and A2-P15, and lesser at points A2-P02, A2-P03, A2-P04, A2-P05, A2-P06 and A2-P07. However, the lowest compliance is at points A2-P14 and A2-P15. Despite the significant difference, the measured settlement and the calculated settlement (the modulus determined by Method 2) have the highest values near points A2-P10 and A2-P11. The discrepancy between the first and the second is a consequence of the simplifications used. Those simplifications are: the external load stresses in the peat layer are equal *σ_z_*=σ_0_=const; the influence of the load from other design areas is not taken into account (first model); the Boussinesq theory and the principle of superposition are applied; the distribution in the organic soil layer is not constant (second model); organic soil constrained modulus of elasticity (*E_m_1_* and *E_m_2_*) is constant throughout the layer. Obtaining such high results in the first method causes the creation of a column of organic soil over the entire thickness. These differences result from considering uniaxial deformations of the organic soil layer and the triaxial stress state in the parameter based on the embankment settlement. The proprietary method allows to obtain the results of calculations that are consistent with those observed on site.

Neglecting the effect of horizontal pressures is based on laboratory experiments, which were performed in an oedometer. The research procedure in the oedometer is adapted to involve the horizontal pressure when comparing the results of the proposed model with that of which was performed in the oedometer, where horizontal pressure is not included. This justifies the assumption that Model 1 gives results of *E* values close to that which derives from the oedometer. This was the reason that the authors, as a first step, consider the module while neglecting the effect of the horizontal pressure.

The lower the constrained modulus of elasticity, the greater the settlement. Determining the appropriate modulus values affects the designed amount of settlement. Overestimated values of the module may result in underestimating the amount of settlement, which might be more significant. It can be seen that when comparing the full-scale experimental studies to the oedometer studies, the oedometric modulus is greater than that of the modulus determined by both methods.

## 5. Conclusions

Organic soil, as in peat, is a complex medium. Therefore, the methods of determining its parameters were examined to find which best reflect the soil’ s natural state, susceptibility, and variables. Full-scale experimental research was conducted in the field (in practice) and simulations were performed to prove that the proposed models can be used in the practical calculations of peat-constrained modulus of elasticity

The authors proposed a method to optimize the constrained modulus of elasticity of peat material by presenting two methods. The first is based on a constant stress distribution, such as in the oedometer test, and the second considers the influence of the loaded area. Both methods were verified numerically based on field experiments on a natural scale and then used to determine the constrained modulus of elasticity of peat material for a real case. The modulus for both methods was determined for peat from the Szczecin area (Poland) based on the described method. 

The methods recommended for the settlement calculation are based on the value of the constrained modulus of elasticity. These methods are based on the assumption of uniaxial settlement. The experimental methods use the uniaxial compression method, which considers the 3-axis stress state. When determining the constrained modulus of elasticity, the presented method takes this fact into account. Using this allows engineers to determine the settlements that occur on site. 

The field-determined constrained modulus of elasticity is lower than the oedometric modulus coming from laboratory tests, which may indicate the subsequent oversizing of the settlement when using the oedometer-based method. If the constrained modulus of elasticity is lower, the settlement is more significant.

Based on the comparison of the measured and calculated settlement values, it can be seen that there is a need to expand Model 2 with additional variables. It is currently the subject of the authors’ research on the issue of the peat compressibility modulus.

## Figures and Tables

**Table 1:** Values of total settlement of the embankment at individual points.

Point No	*s_t_* (m)	Point No	*s_t_* (m)	Point No	*s_t_* (m)
0.0	0.12	0.3	0.25	0.6	0.21
1.0	0.16	1.3	0.33	1.6	0.27
2.0	0.21	2.3	0.37	2.6	0.33
3.0	0.25	3.3	0.43	3.6	0.37
4.0	0.28	4.3	0.45	4.6	0.40
5.0	0.25	5.3	0.43	5.6	0.37
6.0	0.21	6.3	0.37	6.6	0.33
7.0	0.16	7.3	0.33	7.6	0.27
8.0	0.12	8.3	0.25	8.6	0.21
0.1	0.16	0.4	0.28	0.7	0.16
1.1	0.21	1.4	0.35	1.7	0.21
2.1	0.27	2.4	0.39	2.7	0.27
3.1	0.33	3.4	0.45	3.7	0.33
4.1	0.35	4.4	0.48	4.7	0.35
5.1	0.33	5.4	0.45	5.7	0.33
6.1	0.27	6.4	0.39	6.7	0.27
7.1	0.21	7.4	0.35	7.7	0.21
8.1	0.16	8.4	0.28	8.7	0.16
0.2	0.21	0.5	0.25	0.8	0.12
1.2	0.27	1.5	0.33	1.8	0.16
2.2	0.33	2.5	0.37	2.8	0.21
3.2	0.37	3.5	0.43	3.8	0.25
4.2	0.40	4.5	0.45	4.8	0.28
5.2	0.37	5.5	0.43	5.8	0.25
6.2	0.33	6.5	0.37	6.8	0.21
7.2	0.27	7.5	0.33	7.8	0.16
8.2	0.21	8.5	0.25	8.8	0.12

**Table 2:** The results of the constrained modulus of elasticity of the peat materials at the calculation point are determined by the first method.

Point No	*E_m_1_* (kPa)	Point No	*E_m_1_* (kPa)	Point No	*E_m_1_* (kPa)
0.0	2485.33	0.3	1192.96	0.6	1420.19
1.0	1864.00	1.3	903.76	1.6	1104.59
2.0	1420.19	2.3	806.05	2.6	903.76
3.0	1192.96	3.3	693.58	3.6	806.05
4.0	1065.14	4.3	662.76	4.6	745.60
5.0	1192.96	5.3	693.58	5.6	806.05
6.0	1420.19	6.3	806.05	6.6	903.76
7.0	1864.00	7.3	903.76	7.6	1104.59
8.0	2485.33	8.3	1192.96	8.6	1420.19
0.1	1864.00	0.4	1065.14	0.7	1864.00
1.1	1420.19	1.4	852.11	1.7	1420.19
2.1	1104.59	2.4	764.72	2.7	1104.59
3.1	903.76	3.4	662.76	3.7	903.76
4.1	852.11	4.4	621.33	4.7	852.11
5.1	903.76	5.4	662.76	5.7	903.76
6.1	1104.59	6.4	764.72	6.7	1104.59
7.1	1420.19	7.4	852.11	7.7	1420.19
8.1	1864.00	8.4	1065.14	8.7	1864.00
0.2	1420.19	0.5	1192.96	0.8	2485.33
1.2	1104.59	1.5	903.76	1.8	1864.00
2.2	903.76	2.5	806.05	2.8	1420.19
3.2	806.05	3.5	693.58	3.8	1192.96
4.2	745.60	4.5	662.76	4.8	1065.14
5.2	806.05	5.5	693.58	5.8	1192.96
6.2	903.76	6.5	806.05	6.8	1420.19
7.2	1104.59	7.5	903.76	7.8	1864.00
8.2	1420.19	8.5	1192.96	8.8	2485.33

**Table 3:** The values of the *J_i_* parameter in calculation points.

Point No	*J_i_* (kN/m)	Point No	*J_i_* (kN/m)	Point No	*J_i_* (kN/m)
0.0	38.82	0.3	54.99	0.6	52.61
1.0	54.52	1.3	81.28	1.6	77.71
2.0	61.21	2.3	90.87	2.6	86.70
3.0	64.52	3.3	95.26	3.6	90.85
4.0	65.53	4.3	96.56	4.6	92.08
5.0	64.52	5.3	95.26	5.6	90.85
6.0	61.21	6.3	90.87	6.6	86.70
7.0	54.52	7.3	81.28	7.6	77.71
8.0	38.83	8.3	54.99	8.6	52.61
0.1	48.12	0.4	55.74	0.7	48.12
1.1	70.65	1.4	82.40	1.7	70.65
2.1	78.64	2.4	92.18	2.7	78.64
3.1	82.42	3.4	96.65	3.7	82.42
4.1	83.55	4.4	97.97	4.7	83.55
5.1	82.42	5.4	96.65	5.7	82.42
6.1	78.64	6.4	92.18	6.7	78.64
7.1	70.65	7.4	82.40	7.7	70.65
8.1	48.12	8.4	55.74	8.7	48.12
0.2	52.61	0.5	54.99	0.8	38.82
1.2	77.71	1.5	81.28	1.8	54.52
2.2	86.70	2.5	90.87	2.8	61.21
3.2	90.85	3.5	95.26	3.8	64.52
4.2	92.08	4.5	96.56	4.8	65.53
5.2	90.85	5.5	95.26	5.8	64.52
6.2	86.70	6.5	90.87	6.8	61.21
7.2	77.71	7.5	81.28	7.8	54.52
8.2	52.61	8.5	54.99	8.8	38.83

**Table 4:** The results of the constrained modulus of elasticity of the peat materials at the calculation points are determined by the second method.

Point No	*E_m_2_* (kPa)	Point No	*E_m_2_* (kPa)	Point No	*E_m_2_* (kPa)
0.0	323.53	0.3	219.94	0.6	250.52
1.0	340.72	1.3	246.32	1.6	287.81
2.0	291.47	2.3	245.61	2.6	262.73
3.0	258.07	3.3	221.54	3.6	245.54
4.0	234.03	4.3	214.58	4.6	230.21
5.0	258.07	5.3	221.54	5.6	245.54
6.0	291.47	6.3	245.61	6.6	262.73
7.0	340.72	7.3	246.32	7.6	287.81
8.0	323.53	8.3	219.95	8.6	250.52
0.1	300.78	0.4	199.07	0.7	300.78
1.1	336.41	1.4	235.42	1.7	336.41
2.1	291.27	2.4	236.37	2.7	291.27
3.1	249.75	3.4	214.79	3.7	249.75
4.1	238.72	4.4	204.11	4.7	238.72
5.1	249.75	5.4	214.79	5.7	249.75
6.1	291.27	6.4	236.37	6.7	291.27
7.1	336.41	7.4	235.42	7.7	336.41
8.1	300.78	8.4	199.07	8.7	300.78
0.2	250.52	0.5	219.94	0.8	323.53
1.2	287.81	1.5	246.32	1.8	340.72
2.2	262.73	2.5	245.61	2.8	291.47
3.2	245.54	3.5	221.54	3.8	258.07
4.2	230.21	4.5	214.58	4.8	234.03
5.2	245.54	5.5	221.54	5.8	258.07
6.2	262.73	6.5	245.61	6.8	291.47
7.2	287.81	7.5	246.32	7.8	340.72
8.2	250.52	8.5	219.95	8.8	323.53

**Table 5:** Input parameters for determining the constrained modulus of elasticity of peat material by loading it with an embankment.

Point No	*s_t_* (m)	Point No	*s_t_* (m)
A2-P01	0.513	A2-P10	1.175
A2-P02	0.421	A2-P11	1.120
A2-P03	0.327	A2-P12	0.261
A2-P04	0.307	A2-P13	0.102
A2-P05	0.461	A2-P14	0.472
A2-P06	0.371	A2-P15	0.457
A2-P07	0.408	A2-P16	0.179
A2-P08	0.626	A2-P17	0.092
A2-P09	0.247		

**Table 6:** The results of the constrained modulus of elasticity of the peat materials for full-scale experiment at the calculation points determined by the first method.

Point No	*E_m_1_* (kPa)	Point No	*E_m_1_* (kPa)
A2-P01	614.04	A2-P10	268.09
A2-P02	748.22	A2-P11	281.25
A2-P03	963.30	A2-P12	1206.90
A2-P04	1026.06	A2-P13	3088.24
A2-P05	683.30	A2-P14	667.32
A2-P06	849.06	A2-P15	689.28
A2-P07	772.06	A2-P16	1759.78
A2-P08	503.19	A2-P17	3423.91
A2-P09	1275.30		

**Table 7:** The results of the *J_i_* parameter for full-scale experiment at the calculation points.

Point No	*J_i_* (kN/m)	Point No	*J_i_* (kN/m)
A2-P01	204.02	A2-P10	272.78
A2-P02	218.39	A2-P11	277.35
A2-P03	221.59	A2-P12	278.34
A2-P04	222.31	A2-P13	254.45
A2-P05	273.21	A2-P14	253.63
A2-P06	272.27	A2-P15	249.96
A2-P07	267.99	A2-P16	232.61
A2-P08	247.84	A2-P17	255.62
A2-P09	251.70		

**Table 8:** Value of the constrained modulus of elasticity in Szczecin on the Ostrów Brdowski island by the second method.

Point No	*E_m_2_* (kPa)	Point No	*E_m_2_* (kPa)
A2-P01	397.69	A2-P10	232.16
A2-P02	518.74	A2-P11	247.63
A2-P03	677.63	A2-P12	1066.42
A2-P04	724.14	A2-P13	2494.58
A2-P05	592.64	A2-P14	537.36
A2-P06	733.89	A2-P15	546.96
A2-P07	656.84	A2-P16	1299.50
A2-P08	395.91	A2-P17	2778.44
A2-P09	1019.03		

## Data Availability

The data used for the research are included in the report [34]. The company ST3 Offshore consented to the use of the report for research purposes.

## Abbreviations

*A*	analyzed point in models:
*A_i_, B_i_, C_i_, D_i_, E_i_, F_i_*	parameters use to calculate *J_i_*;
*B*	embankment width (m);
*E*	the constrained modulus of elasticity (kPa);
*E_m_1_*	constrained modulus of elasticity of peat material in the column under consideration, determined by the first model (kPa);
*E_m_2_*	modulus of compressibility in the considered column determined by the second model (kPa);
*h_0_*	primary thickness of soil sample in oedometer test (m);
*h_n_*	the height of the embankment (m);
*H_T_*	thickness of the organic soil (m);
*J_i_*	parameter includes influens of organic soil thikness and dimension of embankment in stresses (kN/m);
*l*	a distance of the force from the analyzed point in the embankment (m);
*L*	embankment length (m);
*m_v_*	the coefficient of volume compressibility (m^2^/kN);
*M_0_*	the oedometer constrained modulus of elasticity (kPa);
*P*	the force acting on the subsoil in the analyzed calculation area (kN)
*s*	settlement of the soil sample in oedometer test (m);
*w*	moisture of soil (%);
*x_A_, y_A_*	the coordinates of the analyzed point (m);
*x*	axis of width;
*y*	axis of length;
*z*	depth consideration space (m);
*z*	axis of depth;
*z_A_*	axis of depth
*γ*	the unit weight of the embankment (kN m^−3^);
*Δσ_i_*	stress increment (kPa);
*ε*	unit deformation of the sample;
*ρ*	density of soil (tm^−3^);
*ρ_s_*	specific gravity (tm^−3^);
*σ’_p_*	the initial pre-consolidation pressure (kPa);
*σ_0_*	loaded with the embankment (kPa);
*σ_0_*	compressive load on the soil sample in oedometer test (kPa);
*σ_z_*	stress in the soil (kPa).
